# Triglyceride-glucose index: a novel evaluation tool for all-cause mortality in critically ill hemorrhagic stroke patients-a retrospective analysis of the MIMIC-IV database

**DOI:** 10.1186/s12933-024-02193-3

**Published:** 2024-03-18

**Authors:** Yongwei Huang, Zongping Li, Xiaoshuang Yin

**Affiliations:** 1 Department of Neurosurgery, School of Medicine, Mianyang Central Hospital, University of Electronic Science and Technology of China, Mianyang, Sichuan China; 2 Department of Immunology, School of Medicine, Mianyang Central Hospital, University of Electronic Science and Technology of China, Mianyang, Sichuan China

**Keywords:** Triglyceride-glucose index, Hemorrhagic stroke, MIMIC-IV database, All-cause mortality, Prognosis

## Abstract

**Background:**

Hemorrhagic stroke (HS), including non-traumatic intracerebral hemorrhage (ICH) and subarachnoid hemorrhage (SAH), constitutes a substantial proportion of cerebrovascular incidents, accounting for around 30% of stroke cases. The triglyceride-glucose index (TyG-i) represents a precise insulin resistance (IR) indicator, a crucial metabolic disturbance. Existing literature has demonstrated an association between TyG-i and all-cause mortality (ACM) among individuals suffering from ischemic stroke (IS). Yet, the TyG-i prognostic implications for severe HS patients necessitating intensive care unit (ICU) admission are not clearly understood. Considering the notably elevated mortality and morbidity associated with HS relative to IS, investigating this association is warranted. Our primary aim was to investigate TyG-i and ACM association among critically ill HS patients within an ICU context.

**Methods:**

Herein, patients with severe HS were identified by accessing the Medical Information Mart for Intensive Care-IV (MIMIC-IV, version 2.2) database, using the International Classification of Diseases (ICD)-9/10 as diagnostic guidelines. Subsequently, we stratified the subjects into quartiles, relying on their TyG-i scores. Moreover, we measured mortality at ICU, in-hospital, 30 days, 90 days, and 1 year as the outcomes. Cox proportional hazards regression analysis and restricted cubic splines (RCS) were deployed for elucidating the relation between the TyG-i and ACM while utilizing the Kaplan-Meier (K-M) method to estimate survival curves. The findings’ robustness was assessed by conducting subgroup analysis and interaction tests employing likelihood ratio tests.

**Results:**

The analysis included 1475 patients, with a male predominance of 54.4%. Observed mortality rates in the ICU, hospital, 30 days, 90 days, and 1 year were 7.3%, 10.9%, 13.8%, 19.7%, and 27.3%, respectively. Multivariate Cox regression analysis results manifested that heightened TyG-i was significantly related to ACM at 30 days (adjusted hazard ratio [aHR]: 1.32; 95% confidence interval [CI]: 1.05–1.67; *P* = 0.020), 90 days (aHR: 1.27; 95% CI: 1.04–1.55; *P* = 0.019), and 1 year (aHR: 1.22; 95% CI: 1.03–1.44; *P* = 0.023). The results of RCS analysis demonstrated a progressive elevation in ACM risk with rising TyG-i levels. Interaction tests found no significant effect modification in this relationship.

**Conclusion:**

In summary, TyG-i exhibits a significant correlation with ACM among patients enduring critical illness due to HS. This correlation underscores the probable utility of TyG-i as a prognostic tool for stratifying HS patients according to their risk of mortality. Applying TyG-i in clinical settings could enhance therapeutic decision-making and the management of disease trajectories. Additionally, this investigation augments existing research on the linkage between the TyG-i and IS, elucidating the TyG-i’s role in predicting mortality across diverse stroke categories.

**Supplementary Information:**

The online version contains supplementary material available at 10.1186/s12933-024-02193-3.

## Introduction

Stroke is the second leading cause of mortality globally and the foremost cause of long-term disability, as stated by the World Health Organization (WHO) [1]. Hemorrhagic stroke (HS), comprising intracerebral hemorrhage (ICH) within the brain tissue and spontaneous subarachnoid hemorrhage (SAH) in the subarachnoid space, is particularly severe, contributing to 10-20% of all stroke cases and over 40% of stroke-related deaths [1–3]. While current HS management strategies include supportive care, the effectiveness of interventions such as complication prevention, hemostatic and anti-hypertensive therapies, and surgery in reducing primary brain injury is not well-established [4, 5]. This is particularly true for critical HS patients, where treatment outcomes can significantly vary. For instance, SAH resulting from intracranial aneurysm rupture can achieve satisfactory clinical outcomes with appropriate intervention, whereas ICH often leads to severe brain damage and profound neurological complications. Recent advances suggest that a multi-faceted management approach, involving aggressive blood pressure management, glycemic control, temperature management, and anticoagulation adjustment, may improve functional outcomes for patients experiencing acute cerebral hemorrhage [6]. This underscores the need for comprehensive treatment strategies. With the global population aging, the burden of stroke, especially in intensive care units (ICU), is intensifying. Thus, the identification of prognostic indicators that can foresee adverse outcomes in stroke patients is of paramount importance. These indicators should be straightforward, user-friendly, cost-efficient, and readily applicable in clinical settings.

Prognostic factors that can predict adverse outcomes in critical HS patients are vital. The triglyceride-glucose index (TyG-i), a well-recognized indirect marker of insulin resistance (IR), combines fasting blood glucose (FBG) and triglycerides (TG) levels [7–9]. It has been extensively used to evaluate the relationship between lipid metabolism and glycemic status, proving effective in predicting adverse outcomes in cardiovascular diseases [10–15]. Despite TyG-i’s prognostic efficacy in the prediction of recurrences, morbidity, and mortality among IS patients and other conditions has been substantiated [16–19]. the role of TyG-i in predicting clinical outcomes in HS, particularly among critically ill patients, remains unclear.

This study focuses on the TyG-i’s potential as a predictive marker for mortality in critically ill HS patients, a group facing significantly higher risks than those with IS. By analyzing data from the extensive Medical Information Mart for Intensive Care (MIMIC)-IV database, we aim to elucidate the association between TyG-i levels and all-cause mortality (ACM) in this vulnerable population. Our research seeks to fill the gap in knowledge concerning the predictive value of TyG-i for HS patients, aiming to improve healthcare management and facilitate timely intervention strategies for those at elevated risk.

## Materials and methods

### Study population

This retrospective study aimed to examine health-related data acquired by accessing the MIMIC-IV (version 2.2) database that was developed and maintained by the MIT Computational Physiology Laboratory and comprises comprehensive and extensive medical records of patients admitted to Beth Israel Deaconess Medical Center ICUs [20]. Data extraction was conducted by one of the authors, Yongwei Huang, who met the requirements for accessing the database (Record ID: 12,150,448). The author (Yongwei Huang) underwent specialized training to ensure adherence to established protocols and standardized procedures. We developed detailed data extraction steps and conducted trial extractions before the official data extraction phase to test and refine the clarity and operability of these steps. Besides, we employed multiple validation measures to ensure the accuracy of the data. This included independent review of key data points and consistency checks using statistical software to identify and correct possible input errors or inconsistencies.

Herein, we enrolled HS patients on the basis of the International Classification of Diseases (ICD)-9/10 guidelines, including ICD-9 code 431 and ICD-10 codes I610–I619 and I62.9 for ICH, as well as ICD-9 code 430 and ICD-10 codes I60, I600–I6012, I6000–I6002, I6020–I6022, I6030–I6032, and I6050–I6052 for non-traumatic SAH. To uphold the study’s integrity and ensure the robustness of its findings, stringent exclusion criteria were implemented: (1) individuals younger than 16 years at initial admission were omitted; (2) subjects with multiple ICU admissions for HS, with only the inaugural admission’s data being incorporated; (3) individuals diagnosed with advanced-stage renal impairment, hepatic cirrhosis, or malignancies; (4) patients whose duration of stay in the ICU was below three hours; (5) individuals lacking comprehensive data on the day of admission.

**Fig. 1:**
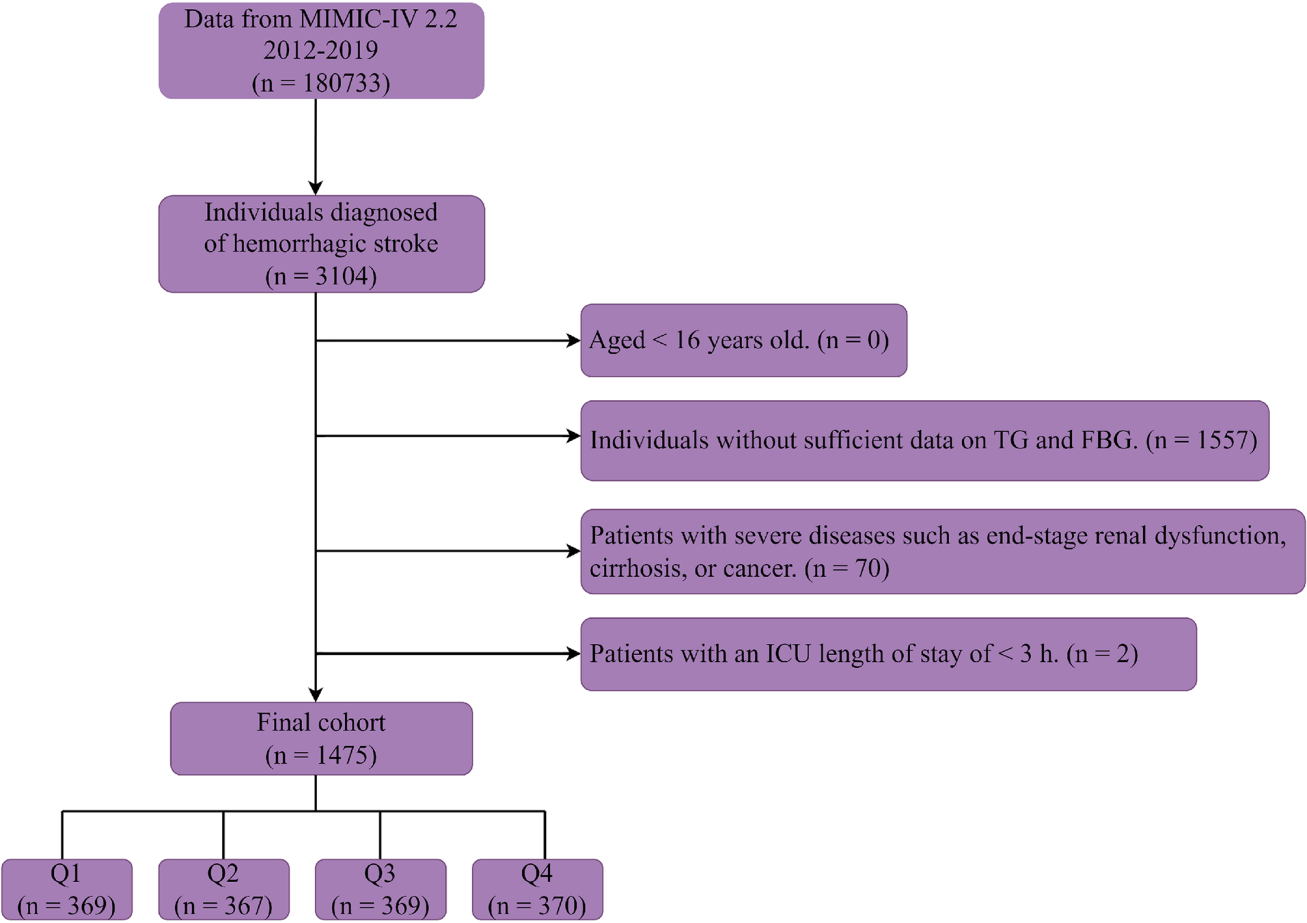
The flow of the enrolled patients throughout the trial. MIMIC-IV: Medical Information Mart for Intensive Care-IV; FBG: fasting blood glucose; TG: triglycerides; ICU: intensive care unit

Subsequent to the application of these criteria, a cohort of 1475 patients was ascertained for inclusion in the analysis (Fig. 1). To dissect the variables pertinent to HS, we stratified the cohort into quartets on the basis of the TyG-i quartiles. The TG and FBG measurement-derived TyG-i represents a critical determinant for evaluating IR and metabolic discrepancies. Through an exhaustive evaluation of the data procured from these participants, this study endeavors to decode the intricate linkage between the TyG-i and HS, thereby furnishing indispensable insights conducive to the formulation of enhanced clinical intervention and prevention modalities.

### Data collection

For data retrieval from the database, PostgreSQL software (version 13.7.2) alongside Navicat Premium (version 16) was deployed. The extraction procedure was facilitated by applying Structured Query Language (SQL). This process targeted the acquisition of data across five principal domains: (1) Demographics encompassing age, sex, and ethnic background. (2) Indices of clinical severity including the Glasgow Coma Scale (GCS), Sequential Organ Failure Assessment (SOFA) score, Simplified Acute Physiology Score (SAPS)-II, Systemic Inflammatory Response Syndrome (SIRS) score, and Oxford Acute Severity of Illness Score (OASIS). (3) Physiological metrics, including, mean blood pressure (MBP), systolic (SBP) and diastolic blood pressure (DBP), heart and respiratory rates, body temperature in degrees Celsius, and oxygen saturation measured via pulse oximetry (SpO_2_). (4) Hematological and biochemical parameters, including hemoglobin concentration (Hb), red blood cell count (RBC), platelet count, white blood cell count (WBC), counts of neutrophils (NEU), monocytes (MON), lymphocytes (LYM), activated partial thromboplastin time (APTT), international normalized ratio (INR), TG, FBG, sodium levels, and serum creatinine. (5) Existing comorbidities such as hypertension, diabetes mellitus, intraventricular hemorrhage (IVH), heart failure (HF), cardiac arrhythmias, peripheral vascular disease (PVD), chronic obstructive pulmonary disease (COPD), renal failure (RF), hepatic disorders, malignant neoplasms, coagulopathy, sepsis, and the Charlson Comorbidity Index (CCI), as well as therapeutic interventions including mechanical ventilation (MT) and vasopressor use.

The observational span for each subject initiated from the point of admission and extended to the event of mortality. TyG-i was calculated as follows: Ln [(TG (mg/dl) × FBG (mg/dl))/2] [17, 18]. The analysis relied on laboratory values and scores that indicated disease severity, which were collected within the first 24 h following ICU admission. To eliminate the missing data influence, variables exhibiting in excess of a 20% absence rate were systematically excluded from the analysis.

### Clinical outcomes

The endpoints of the current study were ICU, in-hospital, 30 days, 90 days, and 1 year ACM. Crucially, the time of death were specified as occurrences of death within a defined period following admission to the ICU, rather than merely identifying whether the patient was deceased at a specific time point.

### Statistical analysis

This study stratified subjects into quartiles relying on their TyG-i values (Q1-Q4). Quantitative variables are reported as mean ± standard deviation (SD) or median and interquartile range (IQR), contingent upon the data distribution while representing the qualitative variables as counts and proportions. Analysis of continuous variables adhering to a normal distribution utilized the t-test or analysis of variance (ANOVA), whereas the Mann-Whitney U test or Kruskal-Wallis test was applied for variables diverging from normality. Pearson’s chi-squared test facilitated the comparison of categorical variables across TyG-i quartiles. The ACM incidence was ascertained for each quartile throughout the observation period.

Moreover, we deployed the Kaplan-Meier (K-M) survival method to ascertain the endpoint incidence rates within groups defined by TyG-i levels, employing the log-rank test to determine the statistical discrepancies. The TyG-i and the study endpoints association was quantified using Cox proportional hazards models, generating hazard ratios (HRs) and 95% confidence intervals (CIs), incorporating three models for the adjustment for confounders: Model 1 (baseline model, not depicted), Model 2 (adjusted for age, ethnicity, and sex), and Model 3 (adjusted covariates in Model 2 and hypertension, diabetes, RF, liver disease, and IVH). Herein, we analyzed TyG-i as a continuous and an ordinal metric, utilizing the initial quartile as the reference. Trend analyses across quartiles employed calculations of P-values.

For a nuanced exploration of the TyG-i’s link to ACM, restricted cubic splines (RCS) and penalized spline techniques were utilized to construct Cox proportional hazards models, pinpointing any nonlinear associations and identifying the optimal threshold value through exhaustive evaluation. Subsequently, a bifurcated Cox model assessed the mortality risk on either side of this threshold. Stratification and interaction analyses dissected the influence of gender, age (either below 65 or 65 and above), presence of diabetes, sepsis, and HF, employing likelihood ratio tests to probe for interactions. HRs in subgroups were the same as adjustment in Model 2.

All analyses mandated a significance threshold of *P* < 0.05 (two-tailed) and were executed via R software (version 4.2.2) alongside SPSS 22.0 (IBM SPSS Statistics, Armonk, NY, USA).

## Results

Herein, 1475 individuals with HS in a critical condition were incorporated, with a median age of 69 years (IQR: 57–79), with males constituting 54.4% (*n* = 803) of the study population. The median TyG-i across the cohort was calculated at 8.8 (IQR: 8.4–9.2). Mortality rates observed included 7.3% for the ICU setting, 10.9% for in-hospital, and extended to 13.8%, 19.7%, and 27.3% over 30 days, 90 days, and 1 year, respectively.

### Baseline characteristics

Table 1 lists the baseline demographic and clinical attributes stratified by the TyG-i quartiles. Subjects were allocated into four quartiles depending upon TyG-i values at the hospital admission time (Q1: 7.1–8.4; Q2: 8.4–8.8; Q3: 8.8–9.2; Q4: 9.2–12.2). The median TyG indices for these quartiles were 8.2 (IQR: 7.9–8.3), 8.6 (IQR: 8.5–8.7), 9.0 (IQR: 8.9–9.1), and 9.5 (IQR: 9.3–9.9) respectively. Individuals in the highest TyG-i quartile had elevated ages, GCS, SIRS, and a higher incidence of hypertension, diabetes, renal and liver diseases, coagulopathy, as well as increased body temperature. Additionally, these patients demonstrated higher counts of RBC, Hb, WBC, NEU, and MONO, along with elevated systemic immune-inflammation index (SII), systemic inflammation response index (SIRI), aggregate index of systemic inflammation (AISI), TG, FBG, and serum creatinine levels. Moreover, there was a more frequent application of MT and vasopressor treatment, accompanied by higher mortality rates in the ICU, in-hospital, and 30 days post-admission, in comparison to their counterparts in lower quartiles.

**Table 1:** Characteristics and outcomes of participants categorized by TyG index

Variables	Overall (*n* = 1475)	Q1 (*n* = 369)	Q2 (*n* = 367)	Q3 (*n* = 369)	Q4 (*n* = 370)	P-valve
TyG index	8.8 (8.4–9.2)	8.2 (7.9–8.3)	8.6 (8.5–8.7)	9.0 (8.9–9.1)	9.5 (9.3–9.9)	**< 0.001**
**Demographic variables**						
Age (y, IQR)	69 (57–79)	73 (61–82)	73 (60–81)	67 (55–77)	65 (54–74)	**< 0.001**
Male (n, %)	803 (54.4%)	189 (51.2%)	199 (54.2%)	217 (58.8%)	198 (53.5%)	0.211
**Ethnicity**						**0.003**
White (n, %)	896(60.8%)	234(63.4%)	230(62.7%)	219(59.4%)	213(57.6%)	
Black (n, %)	185(12.5%)	59(16.0%)	59(13.4%)	47(12.7%)	30(8.1%)	
Mexican American (n, %)	64(4.3%)	12(3.3%)	14(3.8%)	18(4.9%)	20(5.4%)	
Other (n, %)	330(22.4%)	64(17.3%)	74(20.2%)	85(23.0%)	107(28.9%)	
**Clinical severity**						
GCS	15 (14–15)	15 (14–15)	15 (14–15)	15 (14–15)	15 (15–15)	**< 0.001**
SOFA	1 (0–2)	1 (0–2)	0 (0–1)	0 (0–1)	0 (0–2)	0.055
SAPS-II	32 (25–40)	32 (24.5–39.5)	33 (25–40)	31(24–40)	33 (26–41)	0.149
SIRS	2 (2–3)	2 (1.5-3)	2 (2–3)	2(2–3)	3 (2–3)	**< 0.001**
OASIS	30 (25–36)	30 (25–35)	30 (25–35)	30(25–35)	31 (25–36)	0.346
**Comorbidities**						
Hypertension (n, %)	928 (62.9%)	242 (65.6%)	248 (57.6%)	231 (62.6%)	207 (56.0%)	**0.007**
Diabetes (n, %)	387 (26.2%)	60 (16.3%)	70 (19.1%)	104 (28.2%)	153 (41.4%)	**< 0.001**
IVH (n, %)	157 (10.6%)	34 (9.2%)	37 (10.1%)	39 (10.6%)	47 (12.7%)	0.463
HF (n, %)	203 (13.8%)	46 (12.5%)	52 (14.2%)	46 (12.5%)	59 (16.0%)	0.461
Cardiac arrhythmias (n, %)	624 (42.3%)	156 (42.3%)	162 (44.1%)	149 (40.4%)	157 (42.4%)	0.784
PVD (n, %)	61 (4.1%)	10 (2.7%)	18 (4.9%)	14 (3.8%)	19 (5.1%)	0.324
COPD (n, %)	55 (3.7%)	9 (2.4%)	15 (4.1%)	15 (4.1%)	16 (4.3%)	0.508
RF (n, %)	941 (63.8%)	205 (55.6%)	218 (59.4%)	247 (66.9%)	271 (73.2%)	**< 0.001**
Liver disease (n, %)	130 (8.8%)	24 (6.5%)	25 (6.8%)	37 (10.0%)	44 (11.9%)	**0.025**
Malignant tumors (n, %)	313 (21.2%)	83 (22.5%)	84 (22.9%)	82 (22.2%)	64 (17.3%)	0.204
Coagulopathy (n, %)	192 (13.0%)	53 (14.4%)	46 (12.5%)	41 (11.1%)	52 (14.1%)	0.532
Sepsis(n, %)	721 (48.9%)	145 (39.3%)	161 (43.9%)	181 (49.1%)	234 (63.2%)	**< 0.001**
CCI	5 (4–7)	5 (4–7)	5 (4–7)	5 (3–7)	5 (3–7)	0.225
**Vital signs**						
SBP (mmHg)	138 (121–153)	136 (121–149)	140 (123–154)	137.5 (122-152.5)	137 (119–154)	0.077
DBP (mmHg)	74 (63–85)	73 (61–83)	74 (65–86)	75 (65-85.5)	72 (62–87)	0.134
MBP (mmHg)	91 (80–103)	90 (78.5–101)	92 (81–104)	91 (82–102)	92 (79–105)	0.185
Heart rate (beats/min)	81 (71–92)	80(70–91)	80 (70–93)	81 (72–92)	82 (70–93)	0.293
SpO_2_ (%)	98 (96–100)	98 (96–100)	98 (96–100)	98 (96–100)	98 (96–100)	0.268
Temperature (℃)	36.8 (36.5–37.1)	36.8 (36.5–37.1)	36.8(36.4–37.1)	36.8 (36.5–37.1)	36.8 (36.6–37.2)	**0.004**
**Laboratory Parameters**						
RBC (10^9^/L)	4.1 (3.6–4.5)	4.0 (3.5–4.4)	4.1 (3.6–4.5)	4.2 (3.7–4.5)	4.0 (3.5–4.5)	**0.006**
Hb (g/L)	12.2 (10.7–13.5)	11.9 (10.7–13.4)	12.3 (10.9–13.6)	12.4 (11.2–13.6)	12.0 (10.3–13.4)	**0.018**
WBC (10^9^/L)	10.2 (7.8–13.3)	9.3 (7.1–12.1)	10 (7.9–12.3)	10.6 (8.1–13.8)	11.4 (8.4–14.4)	**< 0.001**
NEU (10^9^/L)	7.2 (5-9.2)	6.5 (4.6–8.6)	7.2 (4.9-9)	7.3 (5.3–9.2)	7.8 (5.4–10.1)	**< 0.001**
PLT (10^9^/L)	207 (164–258)	203.5 (160-254.5)	201 (164–254)	213 (166.5–264)	209 (166–260)	0.222
MONO (10^9^/L)	0.7 (0.5–0.9)	0.6 (0.5–0.8)	0.7 (0.5–0.9)	0.8 (0.6-1.0)	0.7 (0.6-1.0)	**0.008**
LYM (10^9^/L)	1.4(1-1.8)	1.4(1-1.8)	1.4 (1.1–1.8)	1.5 (1.1–1.8)	1.4 (1.0-1.8)	0.215
SII	1007.1 (602.6-1677.3)	953.2 (543.8-1541.8)	973.8 (589.9-1597.8)	1012.2 (629.9-1676.5)	1150.5 (637.8-1985.8)	**0.012**
SIRI	3.4 (1.9–5.7)	3.1 (1.7-5)	3.2 (1.8–5.1)	3.5 (1.9–5.9)	3.9 (2-6.8)	**< 0.001**
AISI	686.7 (356.6-1251.8)	633.8 (334.7-1085.8)	620.8 (343.2-1075.2)	742.2 (379.8-1322.7)	785.2 (380.8-1492.1)	**0.002**
TG (mg/dL)	111 (79–157)	66 (55.5–80)	96 (83–111)	130.5 (112.5–155)	202 (157–277)	**< 0.001**
FBG (mg/dL)	114 (97–140)	98 (89–111)	110 (97-129.4)	118 (101–139)	145.8 (117–184)	**< 0.001**
Sodium (mmol/L)	139 (137–142)	139 (137–142)	139 (137–141)	139 (137–142)	139 (137–142)	0.873
Serum creatinine	0.9 (0.7–1.1)	0.9 (0.7–1.1)	0.9(0.7–1.1)	0.9(0.8–1.2)	1(0.8–1.3)	**< 0.001**
APTT (s)	12.7(11.7–14.2)	12.6(11.7–14)	12.8(11.7–14.4)	12.7(11.6–13.9)	12.7(11.8–14.3)	0.31
INR	1.1 (1.1–1.3)	1.1 (1.1–1.3)	1.1 (1.1–1.3)	1.1 (1-1.3)	1.1 (1.1–1.3)	0.609
**Treatment**						
MT (n, %)	1038 (70.4%)	228 (61.8%)	251 (68.4%)	264 (71.5%)	295 (79.7%)	**< 0.001**
Vasopressor (n, %)	296 (20.1%)	51 (13.8%)	59 (16.1%)	75 (20.3%)	111 (30.0%)	**< 0.001**
**Outcomes**						
ICU ACM	107 (7.3%)	20 (5.4%)	20 (5.5%)	24 (6.5%)	43 (11.6%)	**0.002**
In-hospital ACM	161 (10.9%)	30 (8.1%)	39 (10.6%)	33 (8.9%)	59 (16.0%)	**0.003**
30 days ACM (n, %)	204 (13.8%)	50 (13.6%)	51 (13.9%)	37 (10.0%)	66 (17.8%)	**0.023**
90 days ACM (n, %)	290 (19.7%)	71 (19.2%)	70 (19.1%)	62 (16.8%)	87 (23.5%)	0.138
1 year ACM (n, %)	403 (27.3%)	99 (26.8%)	94 (25.6%)	96 (26.0%)	114 (30.8%)	0.366

TyG index: Q1: 7.1–8.4; Q2: 8.4–8.8; Q3: 8.8–9.2; Q4: 9.2–12.2

### Clinical outcomes

**Fig. 2:**
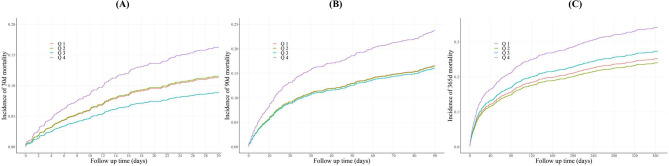
Kaplan-Meier survival analysis curves for (**A**) 30 days, (**B**) 90 days, and (**C**) 1 year ACM. ACM: all-cause mortality

The utilization of K-M survival analysis facilitated the examination of ACM rates across different TyG-i quartiles, as depicted in Fig. 2. It was observed that individuals with elevated TyG-i indices exhibited an increased mortality risk at 30 days, 90 days, and 1 year. However, during the periods of ICU stay and hospitalization, mortality rates did not significantly differ (Figure S1). The relation between the TyG-i and ACM at various intervals (ICU, in-hospital, 30 days, 90 days, and 1 year) was assessed using Cox proportional hazards modeling. The findings indicated the TyG-i as a significant predictor of 30-day mortality in both the initial adjusted model 1 (HR: 1.39; 95% CI: 1.12–1.74; *P* = 0.004) and the comprehensive adjusted model 2 (HR: 1.32; 95% CI: 1.05–1.67; *P* = 0.020) when analyzed as a continuous variable. Conversely, ICU mortality did not exhibit statistical significance (Table S1). Table 2 summarizes the detailed associations between the TyG-i and ACM at 30 days, 90 days and 1 year.

**Table 2:** Cox proportional hazard ratios for ACM at 30 days, 90 days, and 1 year

Categories	Model 1			Model 2		
	HR (95% CI)	P-value	P for trend	HR (95% CI)	P-value	P for trend
30 days mortality						
Continues variable per unit	1.39 (1.12–1.74)	**0.004**		1.32 (1.05–1.67)	**0.020**	
Quartile			**0.003**			**0.014**
Q1 (*N* = 369)	Reference			Reference		
Q2 (*N* = 367)	1.05 (0.71–1.54)	0.821		1.04 (0.70–1.53)	0.857	
Q3 (*N* = 369)	0.84 (0.54–1.29)	0.418		0.79 (0.51–1.22)	0.284	
Q4 (*N* = 370)	1.64 (1.14–2.37)	**0.008**		1.48 (1.01–2.17)	**0.045**	
**90 days mortality**						
Continues variable per unit	1.35 (1.12–1.63)	**0.002**		1.27 (1.04–1.55)	**0.019**	
Quartile			**0.005**			**0.032**
Q1 (*N* = 369)	Reference			Reference		
Q2 (*N* = 367)	1.01 (0.73–1.40)	0.95		1.02 (0.74–1.42)	0.892	
Q3 (*N* = 369)	1.01 (0.72–1.42)	0.964		0.95 (0.67–1.35)	0.783	
Q4 (*N* = 370)	1.60 (1.17–2.18)	**0.003**		1.45 (1.05–2.01)	**0.026**	
**1 year mortality**						
Continues variable per unit	1.28 (1.09–1.51)	**0.002**		1.22(1.03–1.44)	**0.023**	
Quartile			**0.006**			**0.043**
Q1 (*N* = 369)	Reference			Reference		
Q2 (*N* = 367)	0.96 (0.73–1.28)	0.803		0.98 (0.74–1.30)	0.897	
Q3 (*N* = 369)	1.11 (0.84–1.47)	0.453		1.06 (0.79–1.41)	0.718	
Q4 (*N* = 370)	1.50 (1.15–1.97)	**0.003**		1.40 (1.05–1.86)	**0.029**	

HR: hazard ratio; CI: confidence interval; ACM: all-cause mortality. Model 1: adjusted age, sex, and ethnicity; Model 2: adjusted age, sex, ethnicity, hypertension, diabetes, RF, liver disease, and IVH

**Fig. 3:**
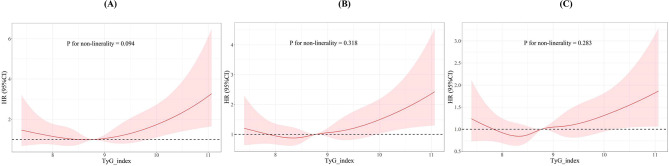
Restricted cubic spline curve for (**A**) 30 days, (**B**) 90 days, and (**C**) 1 year ACM. HR, hazard ratio; CI, confidence interval; TyG index, triglyceride-glucose index; ACM: all-cause mortality

When categorizing the TyG-i as an ordinal variable, individuals in the highest quartile had a significantly increased risk of 30 days ACM in the Cox proportional hazards models: the initial adjusted model 1 (HR: 1.64; 95% CI: 1.14–2.37; *P* = 0.008) and the fully adjusted model 2 (HR: 1.48; 95% CI: 1.01–2.17; *P* = 0.045), relative to those in the lowest quartile, indicating a rising trend in mortality risk with increasing TyG-i levels. This pattern was also mirrored in the multivariate Cox regression analyses concerning 90 days and 1 year mortality rates. However, the association between the highest TyG quartile and increased risk of ICU and in-hospital ACM did not reach statistical significance in the models (Table S1). Additionally, the application of RCS regression modeling demonstrated that mortality risk at 30 days, 90 days, and 1 year escalated in a nonlinear relationship with an ascending TyG-i (P_non−linearity_ = 0.094, P_non−linearity_ = 0.318, and P_non−linearity_ = 0.283, respectively), as illustrated in Fig. 3. Moreover, RCS regression analysis indicated a nonlinear increase in ICU and in-hospital mortality risk with rising TyG-i values (P_non−linearity_ = 0.590, P_non−linearity_ = 0.686, respectively; Figure S2).

### Subgroup analysis

**Fig. 4:**
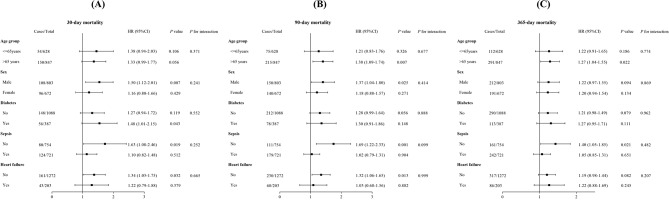
Restricted cubic spline for the association between SHR and length of hospital stay in patients with acute stroke. HR, hazard ratio; CI, confidence interval; ACM: all-cause mortality. HRs were adjusted for age, sex, ethnicity, hypertension, diabetes, RF, liver disease, and IVH

The prognostic utility of the TyG-i for predicting ACM was meticulously evaluated across various patient subgroups, including age, sex, presence of diabetes, sepsis, and HF. The TyG-i was a substantial predictor of elevated 30 days mortality risk within subgroups, notably among males (HR: 1.42; 95% CI: 1.05–1.90) and individuals not afflicted by sepsis (HR: 1.60; 95% CI: 1.13–2.27; Fig. 4A). In a similar vein, for 90 days mortality, a significant correlation was observed with the TyG-i in males (HR: 1.38; 95% CI: 1.105–1.81), individuals aged over 65 years (HR: 1.39; 95% CI: 1.10–1.76), patients without sepsis (HR: 1.69; 95% CI: 1.25–2.29), and patients free from HF (HR: 1.32; 95% CI: 1.05–1.65; Fig. 4B). Furthermore, for the 1 year mortality stratified analysis, the TyG-i was significantly related to an increased mortality risk in individuals older than 65 years (HR: 1.26; 95% CI: 1.02–1.54) and those without sepsis (HR: 1.38; 95% CI: 1.04–1.83; Fig. 4C). Figure S3 represents detailed analyses pertaining to ICU and in-hospital ACM.

## Discussion

Herein, we investigated the correlation between TyG-i and clinical outcomes in a critically ill population with HS from an American cohort, demonstrating that an elevated TyG-i was linked to both short- and longer-term prognosis in those patients. Even after adjusting for the confounding risk factors, TyG-i remained moderately correlated with ACM in the short- and long-term. RCS regression analysis demonstrated “J-shaped” non linear relationship between TyG-i and both short- and long-term ACM. Interaction tests found no significant effect modification in this relationship. Therefore, TyG-i can be a beneficial tool for clinicians in making decisions and could serve as an independent risk factor in critically ill patients with HS.

The TyG-i, a composite measure derived from TG and FGB levels, has been posited as a viable biomarker for metabolic syndromes, atherogenesis, and cardiovascular pathology [18, 21, 22]. Numerous investigations have scrutinized the correlation of TyG-i with cerebrovascular ailments incidence and fatality rates across diverse cohorts. The work of Liu and his team delineated the TyG-i prognostic significance for clinical endpoints in acute IS sufferers with concomitant diabetes mellitus [17], adding the applicability of TyG-i as a predictive indicator. Yang and colleagues showed that higher TyG-i was associated with an augmented risk of neurological decline and mortality [23]. Research by Lee and associates underscored the TyG-i’s utility in forecasting near-term functional outcomes in subjects experiencing acute IS who underwent reperfusion interventions [24]. Within the domain of CAD, the TyG-i has been identified as a potential harbinger of subsequent cardiovascular incidents [25]. An additional investigation, encompassing 5695 subjects, advocated for the surveillance of TyG-i alterations as a method for anticipating detrimental cardiovascular occurrences [26]. Yang et al. conducted a systematic review and meta-analysis, encompassing 592,635 patients, and demonstrated that TyG-i has potential value in optimizing risk stratification for IS in the general population. Furthermore, there is a significant association between high TyG-i and stroke recurrence and high mortality [27]. However, there is still a lack of systematic review of HS, so the study of correlation between TyG-i and the risk and clinical prognosis of HS is quite urgent. A study conducted by Cai et al. [28] elucidated that the TyG-i was significantly correlated with in-hospital and ICU ACM in critically ill patients experiencing IS. However, our investigation did not corroborate this relationship within the cohort of critically ill HS patients. In contrast, we discerned that TyG-i bore an association with ACM at 30 days, 90 days, and 1 year post-event. For critically ill individuals, the prognostic capability for short-term mortality might surpass the relevance of long-term mortality predictions. Yet, for patients who prevail beyond hospital discharge, the utility of an efficacious prognostic indicator for long-term mortality cannot be understated. Our study accentuates the import of TyG-i as an integrative marker in risk stratification, offering a nuanced and comprehensive tool for the precise delineation of individuals at elevated risk. Consequently, this furnishes novel perspectives for the prophylaxis and management of cerebrovascular ailments. Collectively, these inquiries underscore the prospective value of TyG-i as a predictive tool for the clinical trajectories of diseases related to cerebrovascular and cardiovascular health.

Notwithstanding, using TyG-i as a clinical tool has been subjected to scrutiny by various scholars due to the potential confounding effect of hyperglycemia. IR, a condition intimately linked with numerous metabolic syndrome manifestations that include obesity, hyperlipidemia, and hypertension, has been a focal point of investigation. To ascertain its clinical utility, TyG-i has been utilized in assessing IR among individuals deemed at elevated risk within the ambit of extensive clinical investigations. These studies have elucidated that the TyG-i serves as an efficacious instrument for evaluating IR, demonstrating notable predictive accuracy, particularly in cohorts of young and middle-aged individuals [8]. In essence, the TyG-i, under specific circumstances, exhibits superiority over measurements of glucose or TG alone, offering a more comprehensive representation of disease progression [29]. Consequently, the TyG metric has been verified as a robust indicator for a spectrum of cardiovascular and cerebrovascular conditions, alongside other diseases associated with metabolic dysfunctions.

The precise pathophysiological underpinnings delineating the correlation between the TyG-i and the etiology, as well as the progression of cerebrovascular diseases and associated mortality, remain elusive. The linkage is hypothesized to pivot around IR. Research has indicated that while glucose levels may mirror IR originating from hepatic processes, TG levels predominantly reflect IR in adipose tissues, positing the TyG-i as a composite indicator of IR from these dual sources [30]. As an IR marker, the TyG-i is implicated in endothelial dysfunction, inflammatory responses, foam cell formation hastening, and smooth muscle cell proliferation, which are pivotal in atherosclerosis’s nascent stages [31–33]. Miao and colleagues have underscored the link between TyG-i and carotid atherosclerosis severity in IR patients, suggesting its potential as an atherosclerotic biomarker [34]. Ahn and co-researchers have proposed the TyG-i, formulated from lipid and glucose predictors, as a reliable indicator of IR. Further [35], Che and associates have depicted that an augmented TyG-i correlates with a heightened cerebrovascular ailments risk post-adjustment for established confounders [36]. The role of IR extends beyond the genesis of atherogenesis to encompass the progression of advanced plaques by instigating vascular smooth muscle cell apoptosis. TyG-i encapsulates the state of glucose metabolism, inflammatory processes, and oxidative stress [37] and mirrors glycosylation end-product metabolism and platelet activity, potentially leading to endothelial cell-dependent vasodilation. Additionally, elevated TyG-i may signify increased free fatty acid levels, often combined with IR [38–40]. Therefore, mitigating the TyG-i might represent an adjunctive target for individuals predisposed to cerebrovascular conditions. These pathophysiological alterations collectively foster the onset and progression of cerebrovascular disorders, culminating in adverse clinical outcomes.

The principal advantage of this investigation lies in the confirmation that a high TyG-i constitutes a significant independent predictor of increased mortality among critically ill patients with HS within an American cohort. Nonetheless, the study is encumbered by several constraints. Primarily, its retrospective design precludes a definitive determination of causality. Despite the application of multivariate adjustments and subgroup analyses, the potential for residual confounding remains, with certain variables such as subtypes of HS (ICH and SAH), eliminations based on the National Institutes of Health Stroke Scale, stroke onset timing, and specific death causes being inaccessible within the utilized database. Additionally, the other blood fats and lipoproteins are significant confounders. However, upon extraction, missing values for these indicators exceeded 20%. After careful consideration, we decided to exclude these potential confounders from our primary analysis to maintain the integrity and reliability of our findings. Secondarily, the investigation was confined to evaluating the baseline TyG-i without the capability to track its dynamic fluctuations throughout the duration of hospital and ICU admissions. Hence, the necessity to assess the prognostic relevance of alterations in the TyG-i warrants attention in subsequent studies. Tertiarily, the absence of hyperinsulinemic-euglycemic clamp testing within the study framework inhibited assessing the correlation between the TyG-i and IR, benchmarked against the gold standard. This limitation underscores the need for further inquiry to elucidate this association comprehensively.

## Conclusion

In summary, the TyG-i is significantly correlated with ACM among patients enduring critical illness due to HS. This correlation underscores the probable usage of TyG-i as a prognostic means for stratifying HS patients according to their risk of mortality. Applying TyG-i in clinical settings could enhance therapeutic decision-making and the management of disease trajectories. Additionally, this investigation augments existing research on the linkage between the TyG-i and IS, elucidating the TyG-i’s role in predicting mortality across diverse stroke categories.

### Electronic supplementary material

Below is the link to the electronic supplementary material.

Supplementary Material 1

## Data Availability

No datasets were generated or analysed during the current study.

## Abbreviations

TyG index: triglyceride-glucose index
IQR: interquartile range
GCS: Glasgow Coma Scale
SOFA: sequential organ failure assessment
SAPS-II: simplified acute physiological score II
SIRS: systemic inflammatory response syndrome score
OASIS: Oxford acute severity of illness score
IVH: intraventricular hemorrhage
HF: heart failure
PVD: peripheral vascular disease
COPD: chronic obstructive pulmonary disease
RF: renal failure, CCI:Charlson comorbidity index
SBP: systolic blood pressure
DBP: diastolic blood pressure
MBP: mean blood pressure
SpO_2_: saturation of pulse oxygen
RBC: red blood cell
Hb: hemoglobin
WBC: white blood cell
NEU: neutrophils count
PLT: platelet
MON: monocytes count
LYM: lymphocytes count
SII: systemic immune-inflammation index
SIRI: systemic inflammation response index
AISI: aggregate index of systemic inflammation
TG: triglyceride
FBG: fasting blood glucose
APTT: activated partial thromboplastin time
INR: international normalized ratio
MT: mechanical ventilation
ACM: all-cause mortality
