# Supplementary material for: Triglyceride-glucose index: a novel evaluation tool for all-cause mortality in critically ill hemorrhagic stroke patients-a retrospective analysis of the MIMIC-IV database
Source: Cardiovasc Diabetol. 2024 Mar 18;23:100. doi: 10.1186/s12933-024-02193-3 (PMC10949583; doi:10.1186/s12933-024-02193-3)
Supplement: Supplementary file 1 — Supplementary Material 1 [file 12933_2024_2193_MOESM1_ESM.docx]

**Supplemental Materials**

**Supplemental Table 1.** Cox proportional hazard ratios for ACM at ICU and in-hospital.

| **Categories** | **Model 1** | | | **Model 2** | | | **Model 3** | | |
| --- | --- | --- | --- | --- | --- | --- | --- | --- | --- |
|  | **HR (95%CI)** | ***P*-value** | **P for trend** | **HR (95%CI)** | ***P*-value** | **P for trend** | **HR (95%CI)** | ***P*-value** | **P for trend** |
| **ICU mortality** | | | | | | | | | |
| Continues variable Per unit | 1.22(0.92-1.61) | 0.167 |  | 1.32(0.98-1.77) | 0.063 |  | 1.31(0.96-1.79) | 0.092 |  |
| Quartile |  |  | 0.470 |  |  | 0.247 |  |  | 0.394 |
| Q1(N=369) | Reference |  |  | Reference |  |  | Reference |  |  |
| Q2(N=367) | 0.89(0.48-1.63) | 0.698 |  | 0.88(0.48-1.62) | 0.681 |  | 0.92(0.50-1.71) | 0.797 |  |
| Q3(N=369) | 0.87(0.48-1.57) | 0.641 |  | 0.91(0.50-1.67) | 0.769 |  | 0.95(0.52-1.76) | 0.871 |  |
| Q4(N=370) | 1.24(0.72-2.12) | 0.435 |  | 1.39(0.80-2.42) | 0.246 |  | 1.40(0.77-2.54) | 0.273 |  |
| **In-hospital mortality** | | | | | | | | | |
| Continues variable Per unit | 1.21(0.97-1.51) | 0.085 |  | 1.32(1.05-1.66) | **0.016** |  | 1.29(1.01-1.64) | **0.042** |  |
| Quartile |  |  | 0.261 |  |  | 0.153 |  |  | 0.250 |
| Q1(N=369) | Reference |  |  | Reference |  |  | Reference |  |  |
| Q2(N=367) | 1.15(0.73-1.84) | 0.546 |  | 1.17(0.73-1.85) | 0.517 |  | 1.18(0.74-1.89) | 0.483 |  |
| Q3(N=369) | 0.86(0.52-1.41) | 0.544 |  | 0.95(0.57-1.59) | 0.853 |  | 0.93(0.55-1.57) | 0.773 |  |
| Q4(N=370) | 1.32(0.84-2.06) | 0.232 |  | 1.50(0.94-2.37) | 0.09 |  | 1.40(0.87-2.26) | 0.165 |  |

HR: hazard ratio; CI: confidence interval; ACM: all-cause mortality; ICU: intensive care unit. Model 1: Unadjusted; Model 2: Adjusted age, sex, and ethnicity; Model 3: adjusted age, sex, ethnicity, hypertension, diabetes, RF, liver disease, and IVH.

**Supplemental Figure 1.** Kaplan-Meier survival analysis curves for **(A)** ICU and **(B)** in-hospital ACM.

**
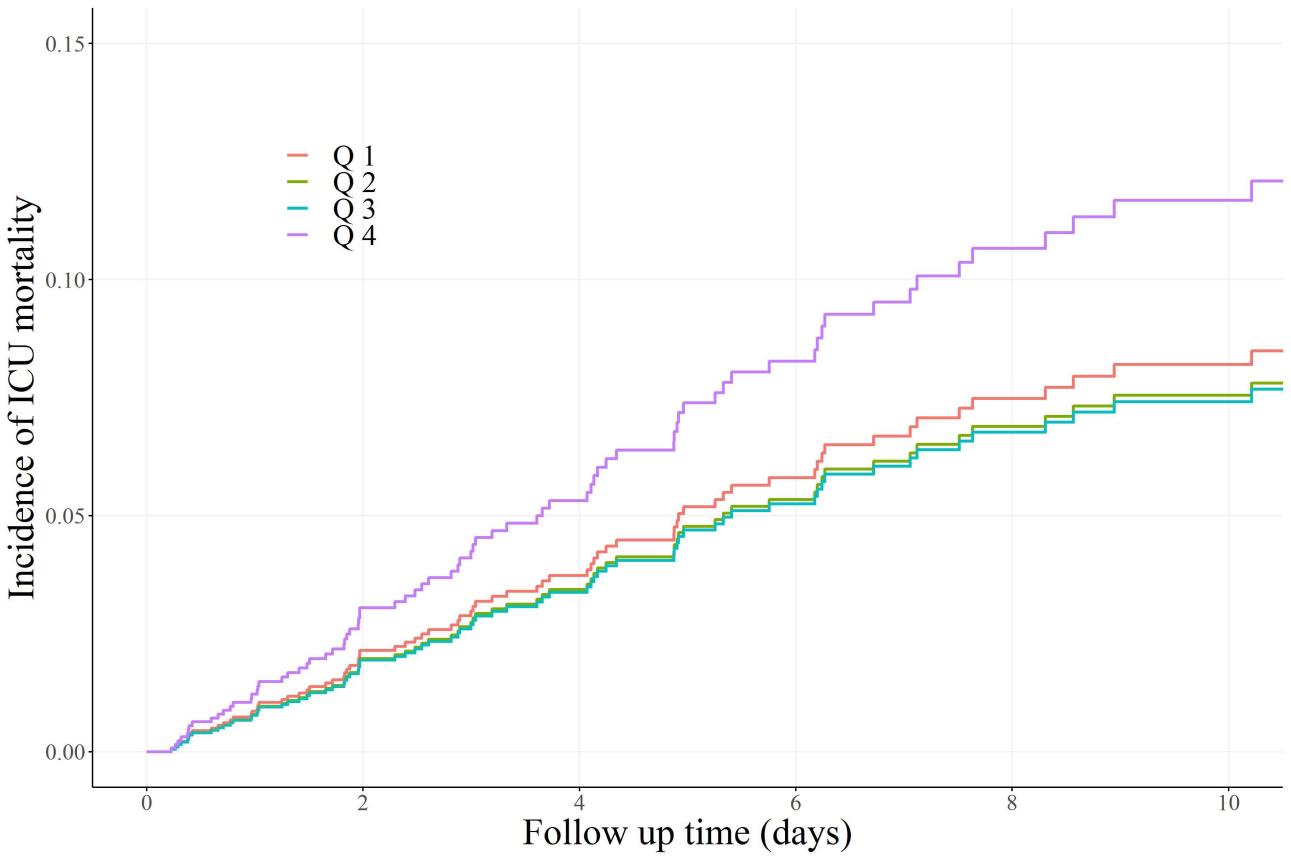

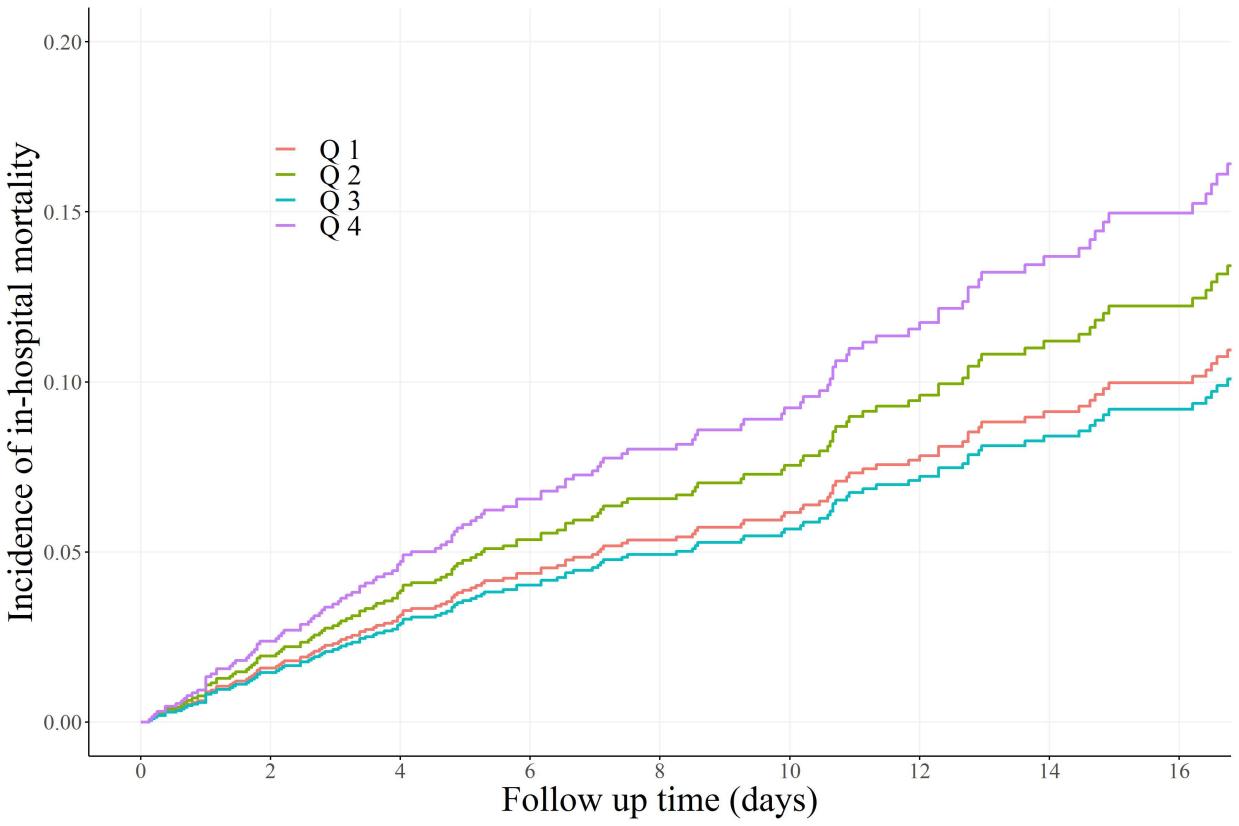
**

1. **(B)**

ACM: all-cause mortality; ICU: intensive care unit.

**Supplemental Figure 2.** Restricted cubic spline curve for **(A)** ICU and **(B)** in-hospital ACM.


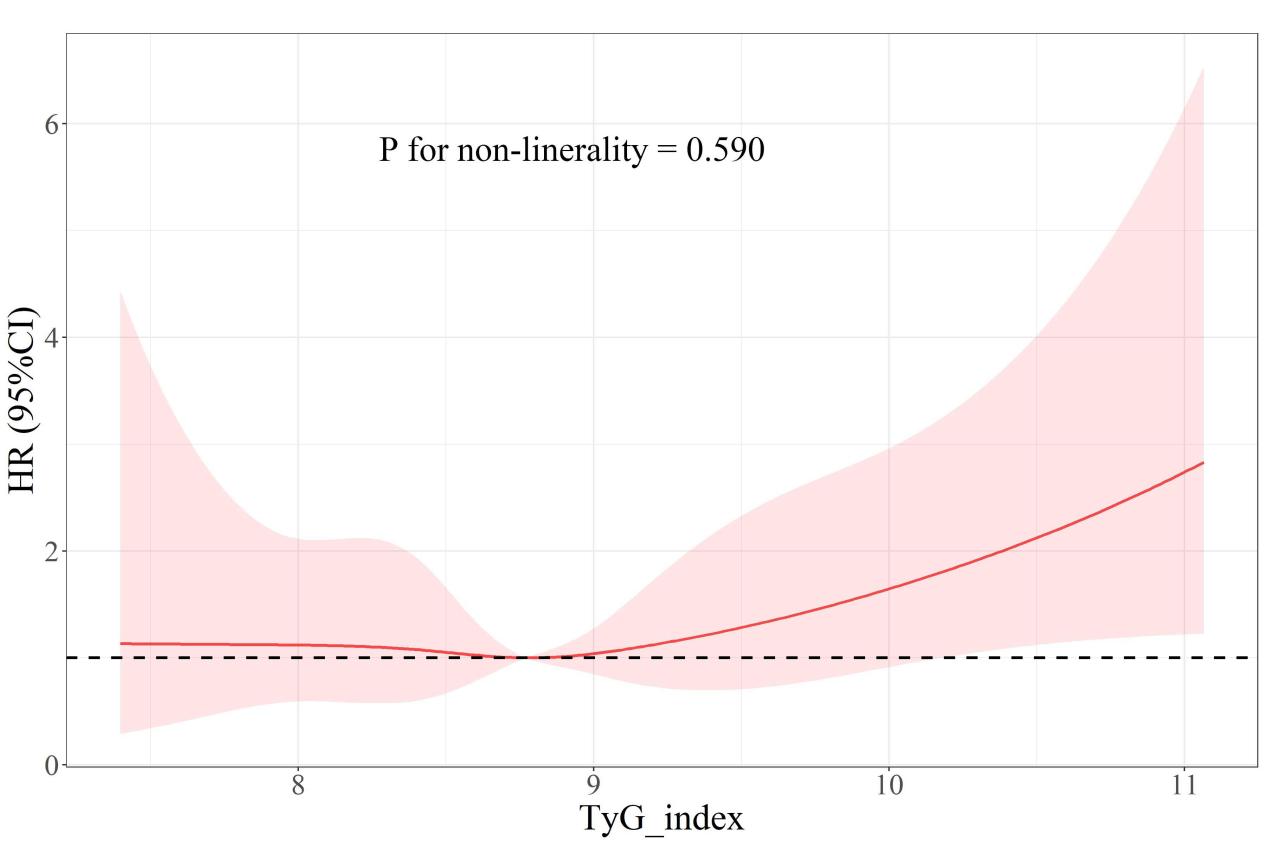

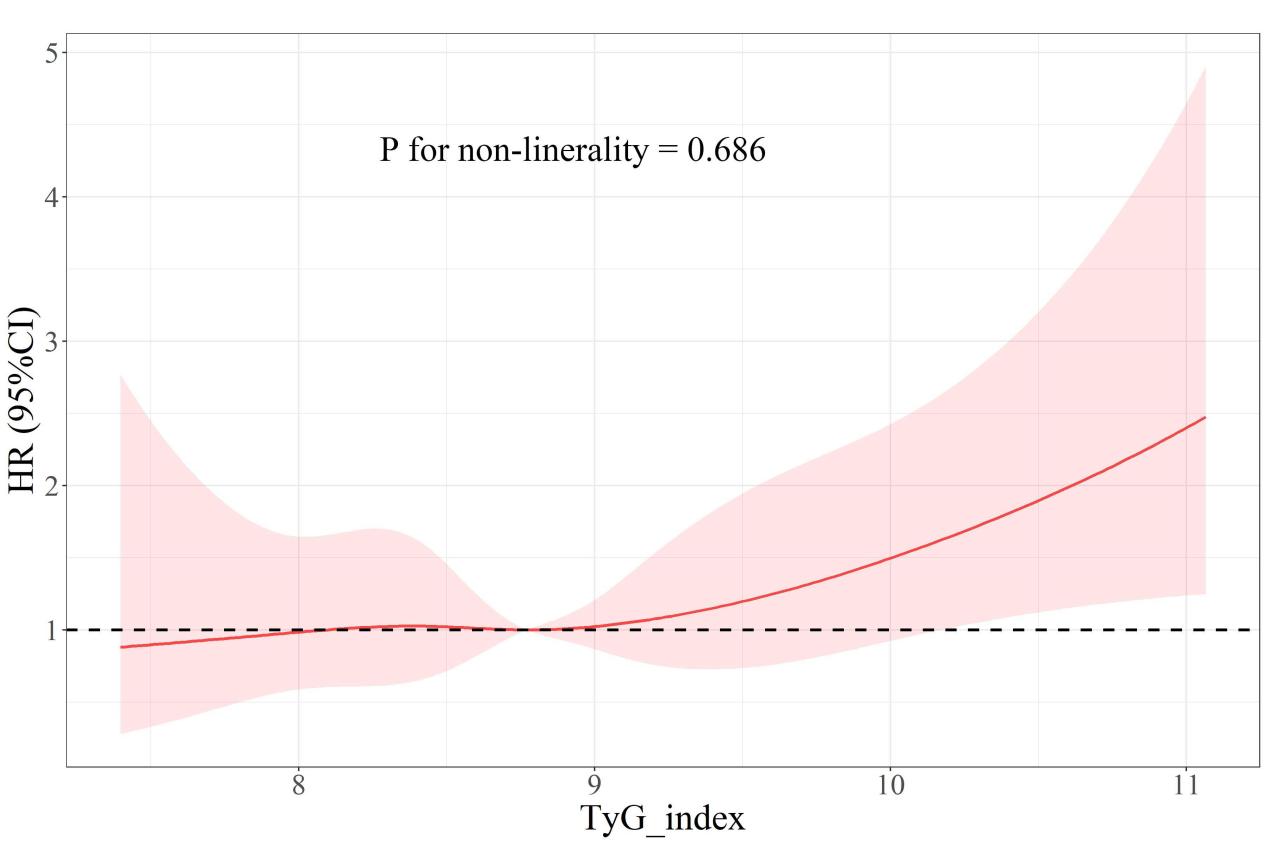


1. **(B)**

HR, hazard ratio; CI, confidence interval; TyG index, triglyceride-glucose index; ACM: all-cause mortality; ICU: intensive care unit.

**Supplemental Figure 3.** Forest plots of hazard ratios for **(A)** ICU and **(B)** in-hospital ACM in different subgroups.


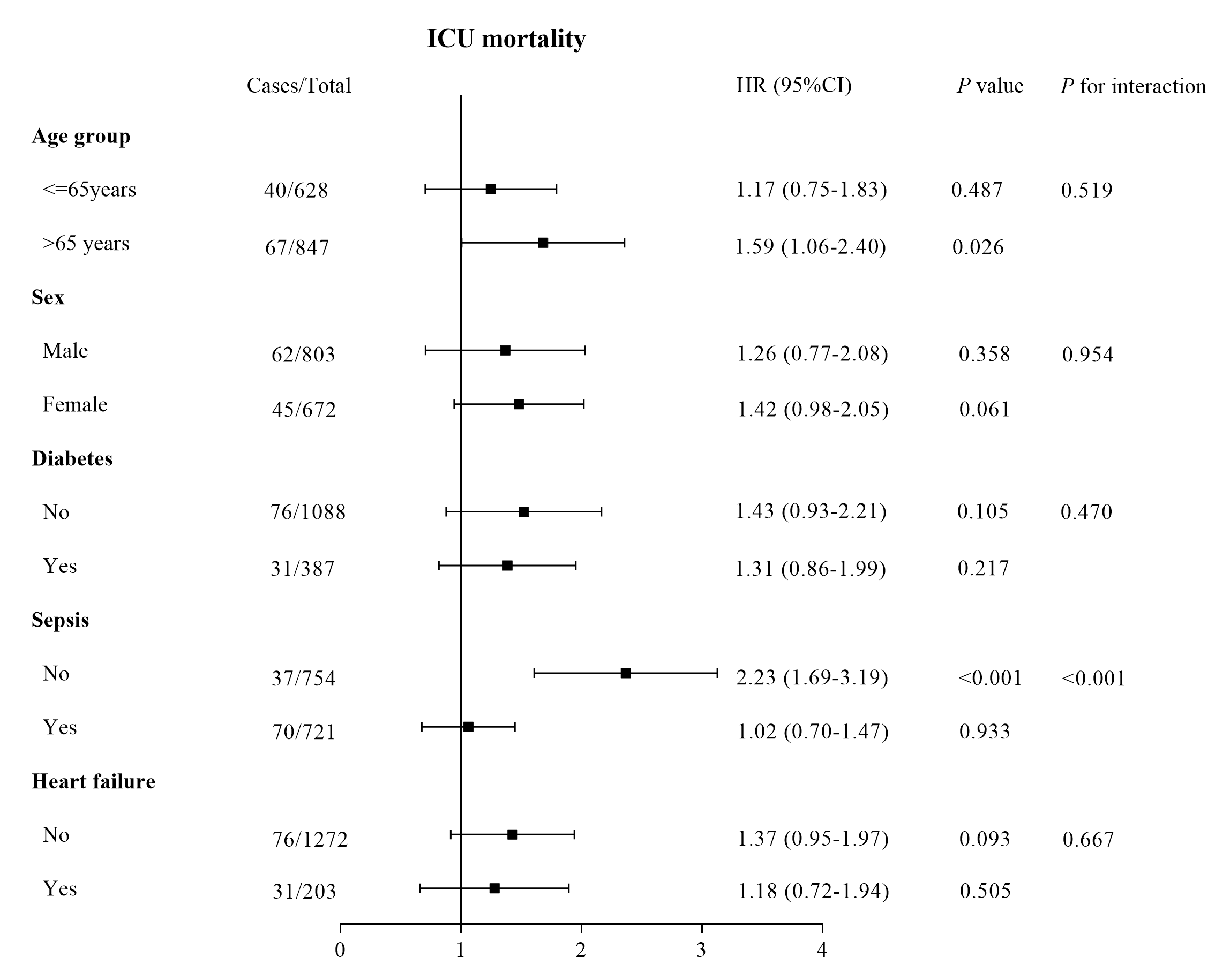

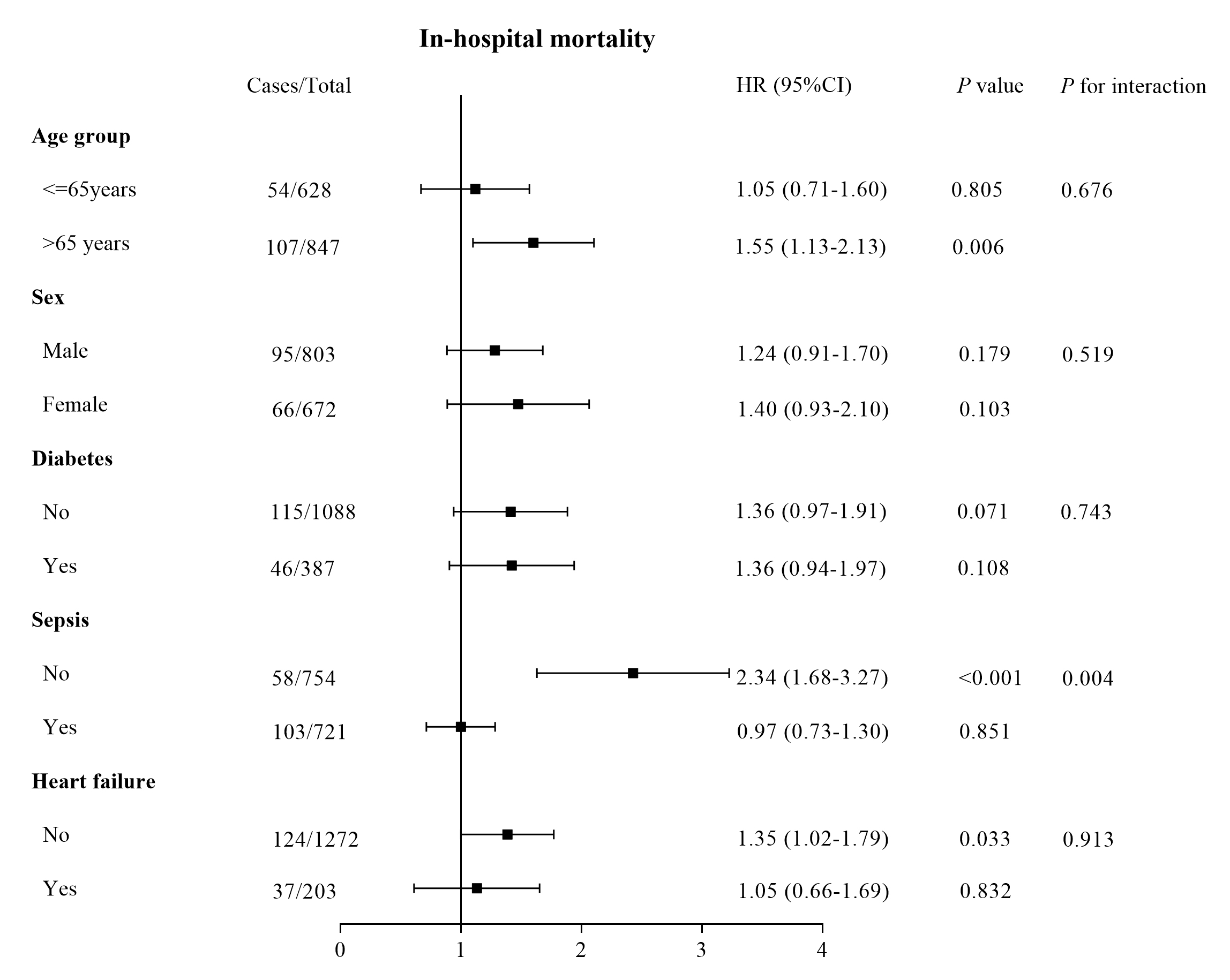


1. **(B)**

HR, hazard ratio; CI, confidence interval; ACM: all-cause mortality. HRs were adjusted for age, sex, ethnicity, hypertension, diabetes, RF, liver disease, and IVH.
